# Puerperal Metro Peritonitis

**Published:** 1869-02-15

**Authors:** J. W. Dora

**Affiliations:** Mattoon, Ills.


### ORIGINAL COMMUNICATIONS.

### *Puerperal Metro Peritonitis.*

BY J. W. DORA, M. D., MATTOON, ILL.

I was summoned Jan. 9th, 1869, to see Mrs. S., aged 30 years, who was three months advanced in pregnancy—the fourth time—she having given birth to two living children and had suffered one abortion ten months previously.

I learned from the patient that she had received a fall some nine days before, from a height of several feet, falling upon the abdomen, which had produced symptoms of approaching abortion. She had suffered pain in the back and loins, with slight chilly sensations, and febrile symptoms, such as headache, loss of appetite, constipation, etc., from the date of the fall.

Upon examination I found a very offensive muco-sanguineous discharge from the uterus, and learned that she had the day previous suffered from severe and protracted rigors, followed by fever. Also several hard chills, with imperfect reaction during that same day previous to my call, with some slight labor pains during the afternoon.

And while I was examining the patient there was a return of the rigors which were protracted more than an hour, presenting all of the alarming symptoms of congestive chills. I found the abdomen very much distended with flatus, very tender and painful; could not endure the slightest pressure, not even the bed clothing to rest heavily upon her; urgent thirst, pulse 120, corded and intermittent during the rigor, but became full, frequent and throbbing. After reaction was partially established, there was hiccough, frequent eructations, retchings and vomiting. Morphine and hot brandy were freely administered during the rigors, and at 8 o'clock P. M. reaction was well established, when fever supplanted the rigors with great violence, the labor pains were augmented, and at 12 M., the embryo was expelled, leaving the placenta within the contracted os uteri, which I succeeded in removing, partially, with the placental hook. The placenta being already decomposed rendered it impossible to remove it entire. The hœmorrhage was slight and principally retained, as the uterus and its appendages, together with the peritoneum were already inflamed, there was, consequently no expulsive force left in the organ to enable it to throw off its putrid contents, but the patient being considerably under the influence of anodynes and stimulants, she rested pretty comfortably for several hours, until the next morning, I adjusted a wide bandage tightly which contributed to her comfort.

10th. Called at 9 o'clock A. M., and found the patient feverish, etc., but I was hopeful that the case would terminate favorably by resolution within a day or two.

I prescribed :

℞. Quinine : gr. xx ;

Sulphite Soda : ʒ ss :

Morphiæ : ℥ j ;

F. & Chart No. 8, Sig., one every two hours, and ordered hop fomentations to the bowels. Called at 3 P. M. and found the symptoms altogether aggravated. I found before me a malignant and well-developed case of Metro-Peritonitis, complicated, as I afterward discovered with Pelvic Cellulitis.

SYMPTOMS AS FOLLOWS :

Tongue coated dark brown, and dry ; thirst insatiable ; pulse 140 per minute, full and bounding ; skin intensely hot ; abdomen tympanitic, tender and painful to the touch, with excruciating throbbing pain low down in the sacral region, from the cellular inflammation, producing that peculiar intolerable tenesmic pain experienced in dysentery. There were profuse, offensive mattery discharges from the bowels at this time, which changed in a few days to dysenterio-muco-purulent character of discharges which were frequent and still very painful. There was head ache, flushed and cyanotic appearance of the countenance, with an anxious expression. A very offensive, cadaverous odor attended all the secretions and evacuations; there was also partial suppression of urine, with complete retention—requiring the use of the catheter to remove what little there was secreted.

TREATMENT.

But before I proceed to detail the therapeutic treatment of this case, I wish to ask the very pertinent question, and answer it, as I think, philosophically, viz: What is this puerperal, or metro-peritonitis, from puerperal causes? Is it an original local phlegmasia, and the constitutional disturbances simply effects, or is its starting point in the blood, a septicemia and the local lesions merely results? To be brief, as I shall not have space to discuss this question "in extenso," I am of the opinion that the disease under consideration is one of blood poisoning. I believe with Meigs, Ferguson, Bedford and others, that puerperal fever is a veritable toxæmia or septicemia, a ferment or morbific matter taken into the circulation by absorption of putrescent matter, generated within the organism of the patient, or conveyed thence through the principle of contagion from a third party by translation or inoculation. It may, therefore be sporadic or epidemic, produced by the absorption into the circulation of decomposing animal matter, contained and retained within the uterus for a period of nine days, perhaps, after decomposition began.

There are, no doubt, in most all cases certain predisposing causes that contribute more or less to develop and excite these morbid influences within the animal economy, such as malarial, miasmatic and idio-miasmatic or any other depressing influence.

We find three indications to be fulfilled in the rational treatment of puerperal fever, viz:

1. The amelioration of the urgent symptoms, or the mitigation of pain and nervous irritation consequent upon the tympanitic distension of the alimentary canal and the local engorgement of the metro-mesenteric vessels pressing upon an inflamed serous membrane.

The second and third indications are to neutralize the materies morbi from the circulating mass, then trust to nature to repair the local lesions which it is generally able to do under favorable circumstances, assisted by restorative treatment and nutritious diet.

In fulfilling the first indication in this case, the bowels having already been evacuated by a dose of compound cathartic pills before I was called, which was followed, as I have before said, by diarrhœa, was to adjust a bandage covering the entire abdomen, extending from the crest of the illium to the epigastrium, maintained in position by an abdominal supporter (Marsh's). And, allow me here to urge the great importance of this simple appliance. It acts like a charm in alleviating that feeling of bursting distention and pain, resulting from the passage of gas from one portion of the canal to another, besides it affords mechanical compression and support which every one knows greatly favors absorption when applied to any portion of the economy where there is an engorgement of its blood vessels. It also presents effusion into the peritoneal cavity; and it does not hinder the application of hot fomentations to the abdomen, at all, which I also kept constantly applied until the active inflammation subsided, after which I applied blister of the cantharidal vesicant over the umbilical and both illiac regions, which, I think, does much to avert local lesions of the ovaries and psoas muscles. I next proceed to the therapeutic treatment, proper in the case, believing, as I have heretofore said, that the case was septicemia. I therefore combined antiseptic with antiphlogestic and eliminative remedies in the beginning. I gave, viz:

℞. Acetate of Potassa: ʒ ij;

P. Aconite Folia: ʒ j;

P. Gelsimin: ʒ ij;

Spts. Nitri Dul.: ℥ jss;

Teaspoonful every two hours, in cold lemon acid, alternated with the following emulsion:

| | | |
|---|---|---|
| ℞. | Sulphite Soda: | ʒ ij; |
| | Quinia Sulph: | ʒ ss; |
| | Tannin: | ʒ xv; |
| | Morph. Sulph: | gr. ij; |
| | Emulsion of Acaciæ: | ℥ ij; |

M. Sig. Teaspoonful every two hours. And ordered the following antiseptic and disinfectant injection both for vagina and rectum.

| | | |
|---|---|---|
| ℞. | Sol Carbolic Acid: | ʒ iv; |
| | Oil Glycerine: | ℥jss; |

M. Add a teaspoonful to a pint of warm water and inject three or four times daily to both the vagina and rectum, to be repeated daily as long as the secretions were offensive.

11th. Nine o'clock A. M. Symptoms unchanged; pulse 150 and weaker; countenance anxious and icterous. Continued the treatment.

12th. Indications much the same. I withdrew the quinine from the emulsion and substituted alum terebinthina sufficient to give 20 drops at a dose every two to four hours.

13th. Prognosis unfavorable. Applied the blisters spoken of to the abdomen, and substituted McMunn's elixir of opium for the morphia in 15 drops doses, in the antiseptic emulsion. Continued the treatment otherwise. The diarrhea at this stage gave place to dysenteric discharges, on account of the extension of inflammation to the colon and rectum, also cellulitis was manifest. Upon examination of the rectum and vagina there could be felt quite a tumor, caused by effusion of sero-purulent matter into the cul de sac situated between the rectum and uterus, which tumor found an outlet some 24 hours afterwards through the walls of the rectum.

14th. Tenesmic pains in the cæcal region are great, not quite as much tympanitis of the bowels, and symptoms more favorable. Treatment continued.

15th. Patient rested well during the night, but no appetite; recommended brandy with warm milk diet, with rare cooked eggs.

16th. Tongue moist and pulse down to 100 per minute, and she can turn herself on to the left side and straighten out the limbs for a few minutes, still thirsty, but has some relish for the milk and eggs.

17th. Pulse 80 and soft, with good volume; slept several hours during the day and night; bowels still tender, and suffers tenesmus, with, at times, severe colicky pains, for which I gave chloroform; emulsion in teaspoonful doses which had a very happy effect, and prescribed:

| | | |
|---|---|---|
| ℞. | Bismuth Subnit: | ʒ ij; |
| | Sulphite Soda: | ʒ ij; |
| | McMunn's Elixir: | ʒ ij; |
| | Emulsion of Acacia | ℥ ij; |

M. Teaspoonful every three or four hours.

18th. Bowels checked up. Continued the prescription, and in addition gave *hydrarg cum cret.* gr. iij, with half teaspoonful of calcined magnesia, night and morning.

19th and 29th. Patient much improved; the bowels act off more healthy, and appetite improving, but still slight irritative fever during the afternoon.

21st and 22d. There is marked improvement in all the symptoms except the rectal tenesmus and urethral irritation. For the latter symptom I prescribed infusion of uva ursi, with teaspoonful doses several times daily of *spts nitre dulcis* and continued the anodyne and antiseptic emulsion.

25th. Convalescence is well established; appetite good; bowels regular; pains abated; urethral irritation much less; diet and stimulants are relished very much.

30th. Patient rapidly improving. Prescribed the following restorative :

℞. Tinc. Cinchi. Comp : ℥ ij ;

Tinc. Gentian : ℥ ij ;

Pyro Phosphate of Iron : ℥ ij ;

Sherry Wine : ℥ viij ;

M. Tablespoonful every four hours and case discharged.

REMARKS.

There was a peculiarity in the fact of there being no return at any time of the lochial discharge, which became suppressed within a few hours after the expulsion of the embryo, no coagula ever passed, and very little uterine discharge of any kind after the fifth day. What became of all that morbid matter is a question; of course, it must have passed off by absorption, or else it found an outlet by way of the pelvic abscess of which I spoke in the early history of the case.

